# Supplementary material for: Influence of Elective Cesarean Calving (with and without Dexamethasone Induction) on the Erythrogram and Iron Serum Profiles in Nellore Calves
Source: Animals (Basel). 2022 Jun 17;12(12):1561. doi: 10.3390/ani12121561 (PMC9219471; doi:10.3390/ani12121561)
Supplement: Supplementary file 1 [file animals-12-01561-s001.zip › animals-1668270-supplementary.pdf]

Table S1 – Mean values and standard error of the mean for the number of Red Blood Cells obtained, expressed in $x10^6mm^3$, from Nellore calves during the first month of life and grouped according to type of birth (elective cesarean section without labor induction, elective cesarean section with labor induction and spontaneous vaginal calving).

RED BLOOD CELLS $X\ 10^6mm^3$				
**Time of life (hours or days)**	Elective cesarean section without inducing labor	Elective cesarean section with labor induction	Spontaneous vaginal calving	*P-Values*
**0 Hours**	7.77 ± 0.29	8.27 ± 0.25	8.39 ± 0.44	0.2170
**3 Hours**	7.36 ± 0.36	8.02 ± 0.28	8.66 ± 0.48	0.0735
**6 Hours**	7.20 ± 0.34	7.84 ± 0.30	8.18 ± 0.45	0.1303
**12 Hours**	6.75 ± 0.31	7.54 ± 0.34	7.59 ± 0.37	0.0294
**1 Day**	6.45 ± 0.31	7.22 ± 0.33	7.32 ± 0.42	0.1401
**2 Days**	6.27 ± 0.30	7.07 ± 0.33	7.21 ± 0.38	0.0654
**3 Days**	6.50 ± 0.36	7.23 ± 0.32	7.06 ± 0.45	0.2172
**5 Days**	6.48 ± 0.37	7.46 ± 0.33	7.10 ± 0.43	0.0582
**7 Days**	6.62 ± 0.31	7.59 ± 0.34	6.84 ± 0.46	0.0456
**10 Days**	7.17 ± 0.33	8.09 ± 0.37	7.70 ± 0.38	0.0602
**15 Days**	7.07 ± 0.28	7.88 ± 0.31	8.04 ± 0.31	0.0480
**30 Days**	7.75 ± 0.43	8.14 ± 0.46	9.06 ± 0.30	0.2913

A, B, C - different letters on the line mean statistically significant difference compared with Tukey's test.

Table S2 – Mean values and standard error of the mean obtained for Hemoglobin, expressed in g/dL, of Nellore calves during the first month of life and grouped according to type of birth (elective cesarean section without labor induction, elective cesarean section with labor induction and spontaneous vaginal calving).

		HEMOGLOBIN g/dL		
**Time of life (hours or days)**	Elective cesarean section without inducing labor	Elective cesarean section with labor induction	Spontaneous vaginal calving	*P-Values*
**0 Hours**	11.29 ± 0.37	11.36 ± 0.46	11.10 ± 0.56	0.4641
**3 Hours**	10.54 ± 0.49	11.08 ± 0.50	11.25 ± 0.59	0.4681
**6 Hours**	10.33 ± 0.46	10.63 ± 0.48	10.65 ± 0.50	0.5407
**12 Hours**	9.71 ± 0.44	10.41 ± 0.52	9.91 ± 0.52	0.2887
**1 Day**	9.36 ± 0.39	10.05 ± 0.52	9.54 ± 0.56	0.3607
**2 Days**	9.06 ± 0.38	9.67 ± 0.54	9.26 ± 0.61	0.2652
**3 Days**	9.19 ± 0.46	9.878± 0.53	9.28 ± 0.76	0.3344
**5 Days**	9.17 ± 0.50	10.22 ± 0.50	9.98 ± 0.73	0.1736
**7 Days**	9.05 ± 0.41	10.35 ± 0.51	10.01 ± 0.55	0.0648
**10 Days**	9.33 ± 0.41^{B}	10.76 ± 0.57^{A}	11.59 ± 0.36^{A}	0.0023
**15 Days**	9.02 ± 0.42^{B}	10.22 ± 0.55^{A}	11.66 ± 0.22^{A}	0.0002
**30 Days**	9.41 ± 0.46^{B}	10.19 ± 0.51^{A}	12.19 ± 0.20^{A}	0.0019

A, B, C - different letters on the line mean statistically significant difference compared with Tukey’s test.

Table S3 – Mean values and standard error of the mean obtained for hematocrit, expressed in %, of Nellore calves during the first month of life and grouped according to type of birth (elective cesarean section without labor induction, elective cesarean section with labor induction and spontaneous vaginal calving).

		HEMATOCRIT %		
**Time of life (hours or days)**	Elective cesarean section without inducing labor	Elective cesarean section with labor induction	Spontaneous vaginal calving	*P-Values*
**0 Hours**	37.65 ± 1.15	39.01 ± 1.73	36.89 ± 1.69	0.1350
**3 Hours**	35.50 ± 1.61	37.52 ± 1.68	37.86 ± 2.10	0.4125
**6 Hours**	34.64 ± 1.54	36.41 ± 1.81	35.80 ± 2.10	0.0384
**12 Hours**	32.11 ± 1.44	34.60 ± 1.54	33.02 ± 1.88	0.2234
**1 Day**	30.59 ± 1.43	32.79 ± 1.79	31.34 ± 1.97	0.4015
**2 Days**	29.02 ± 1.28	31.83 ± 1.89	30.12 ± 1.97	0.1566
**3 Days**	29.25 ± 1.61	32.00 ± 1.76	29.54 ± 2.39	0.2624
**5 Days**	28.56 ± 1.68	32.73 ± 1.70	31.70 ± 2.23	0.0829
**7 Days**	28.46 ± 1.36^{B}	33.01 ± 1.72^{AB}	32.54 ± 1.56^{A}	0.0386
**10 Days**	29.61 ± 1.43^{B}	33.91 ± 1.78^{A}	37.35 ± 1.03^{A}	0.0022
**15 Days**	28.16 ± 1.35^{C}	32.15 ± 1.71^{B}	38.11 ± 0.68^{A}	0.0001
**30 Days**	28.21 ± 1.57^{B}	30.79 ± 1.92^{B}	38.18 ± 0.65^{A}	0.0040

A, B, C - different letters on the line mean statistically significant difference compared with Tukey's test.

Table S4 – Mean values and standard error of the mean obtained for Mean Corpuscular Volume (MCV), expressed in fL, of Nellore calves during the first month of life and grouped according to type of birth (elective cesarean section without labor induction, elective cesarean section with labor induction and spontaneous vaginal calving).

		**MCV** *fL*		
**Time of life (hours or days)**	Elective cesarean section without inducing labor	Elective cesarean section with labor induction	Spontaneous vaginal calving	*P-Values*
**0 Hours**	48.76 ± 0.80	47.04 ± 1.10	44.51 ± 1.80	0.0520
**3 Hours**	48.50 ± 0.80^{A}	46.78 ± 1.03^{AB}	44.14 ± 1.76^{B}	0.0417
**6 Hours**	48.63 ± 0.76^{A}	46.36 ± 1.03^{AB}	44.04 ± 1.72^{B}	0.3862
**12 Hours**	47.79 ± 0.78^{A}	45.79 ± 0.98^{AB}	43.80 ± 1.73^{B}	0.0184
**1 Day**	47.59 ± 0.73^{A}	45.39 ± 1.07^{AB}	43.10 ± 1.64^{B}	0.4015
**2 Days**	46.56 ± 0.79^{A}	44.82 ± 0.98^{B}	41.81 ± 1.52^{B}	0.0114
**3 Days**	45.74 ± 0.79^{A}	44.13 ± 0.95^{AB}	41.70 ± 1.32^{B}	0.0379
**5 Days**	44.22 ± 0.68	43.86 ± 0.90	44.68 ± 1.34	0.9492
**7 Days**	43.08 ± 0.73^{B}	43.50 ± 0.89^{B}	48.60 ± 2.22^{A}	0.0165
**10 Days**	41.42 ± 0.66^{B}	41.99 ± 1.19^{B}	49.41 ± 2.25^{A}	0.0012
**15 Days**	39.81 ± 0.74^{B}	40.66 ± 1.05^{B}	48.03 ± 1.76^{A}	0.0002
**30 Days**	36.70 ± 0.96^{B}	37.90 ± 0.79^{AB}	42.62 ± 1.32^{A}	0.0031

A, B - different letters on the line mean statistically significant difference compared with Tukey's test.

Table S5 – Mean values and standard error of the mean obtained for Mean Corpuscular Hemoglobin (MCH), expressed in pg, of Nellore calves during the first month of life and grouped according to type of birth (elective cesarean section without labor induction, elective cesarean section with labor induction and spontaneous vaginal calving).

		**MCH** *pg*		
**Time of life (hours or days)**	Elective cesarean section without inducing labor	Elective cesarean section with labor induction	Spontaneous vaginal calving	*P-Values*
**0 Hours**	14.56 ± 0.31	13.64 ± 0.25	13.39 ± 0.74	0.1925
**3 Hours**	14.31 ± 0.25	13.74 ± 0.26	13.11 ± 0.61	0.0983
**6 Hours**	14.34 ± 0.24^{A}	13.48 ± 0.22^{AB}	13.10 ± 0.52^{B}	0.0493
**12 Hours**	14.34 ± 0.29^{A}	13.69 ± 0.25^{AB}	13.11 ± 0.58^{B}	0.0117
**1 Day**	14.56 ± 0.29	13.85 ± 0.28	13.13 ± 0.59	0.0576
**2 Days**	14.48 ± 0.26^{A}	13.56 ± 0.24^{AB}	12.78 ± 0.44^{B}	0.0022
**3 Days**	14.38 ± 0.34	13.55 ± 0.24	13.05 ± 0.52	0.0608
**5 Days**	14.20 ± 0.29	13.63 ± 0.19	14.00 ± 0.44	0.5120
**7 Days**	13.66 ± 0.26	13.57 ± 0.25	14.79 ± 0.51	0.0626
**10 Days**	13.01 ± 0.23^{B}	13.26 ± 0.40^{B}	15.24 ± 0.65^{A}	0.0027
**15 Days**	12.70 ± 0.25^{B}	12.86 ± 0.34^{AB}	14.63 ± 0.52^{A}	0.0036
**30 Days**	12.14 ± 0.50	12.44 ± 0.57	13.50 ± 0.39	0.2557

A, B - different letters on the line mean statistically significant difference compared with Tukey's test.

Table S6 – Mean values and standard error of the mean obtained for the Mean Corpuscular Hemoglobin Concentration (MCHC), expressed in %, of Nellore calves during the first month of life and grouped according to type of birth (elective cesarean section without induction of labor, cesarean section elective with labor induction and spontaneous vaginal calving).

		**MCHC %**		
**Time of life (hours or days)**	Elective cesarean section without inducing labor	Elective cesarean section with labor induction	Spontaneous vaginal calving	*P-Values*
**0 Hours**	29.94 ± 0.32	29.16 ± 0.26	30.03 ± 0.61	0.1665
**3 Hours**	29.63 ± 0.36	29.49 ± 0.21	29.73 ± 0.40	0.7276
**6 Hours**	29.78 ± 0.32	29.24 ± 0.30	29.93 ± 0.86	0.3862
**12 Hours**	30.24 ± 0.34	30.20 ± 0.33	30.03 ± 0.42	0.5557
**1 Day**	30.71 ± 0.48	30.69 ± 0.31	30.50 ± 0.35	0.9348
**2 Days**	31.22 ± 0.21	30.44 ± 0.29	30.68 ± 0.24	0.0703
**3 Days**	31.49 ± 0.37	30.90 ± 0.21	31.40 ± 0.48	0.4103
**5 Days**	32.18 ± 0.37^{A}	31.16 ± 0.33^{B}	31.38 ± 0.23^{AB}	0.0201
**7 Days**	31.83 ± 0.36	31.36 ± 0.31	30.63 ± 0.36	0.0757
**10 Days**	31.55 ± 0.25	31.69 ± 0.36	30.95 ± 0.25	0.3134
**15 Days**	32.07 ± 0.38^{A}	31.75 ± 0.26^{A}	30.53 ± 0.23^{B}	0.0071
**30 Days**	33.17 ± 0.83	32.89 ± 1.07	31.77 ± 0.18	0.6246

A, B - different letters on the line mean statistically significant difference compared with Tukey’s test.

Table S7 – Mean values and standard error of the mean obtained for mean Serum Iron (SFe), expressed in µL/dL, of Nellore calves during the first month of life and grouped according to type of birth (elective cesarean section without labor induction, elective cesarean section with labor induction and spontaneous vaginal calving).

		SFe µL/dL		
**Time of life (hours or days)**	Elective cesarean section without inducing labor	Elective cesarean section with labor induction	Spontaneous vaginal calving	*P-Values*
**0 Hours**	154.46 ± 16.17^{A}	140.80 ± 11.93^{A}	65.32 ± 9.19^{B}	0.0004
**3 Hours**	122.72 ± 15.83^{A}	103.39 ± 10.62^{AB}	66.36 ± 7.13^{B}	0.0101
**6 Hours**	78.93 ± 13.27	66.18 ± 8.32	55.77 ± 7.01	0.3452
**12 Hours**	50.55 ± 8.12	46.06 ± 10.65	41.22 ± 5.82	0.8046
**1 Day**	38.40 ± 7.18	49.11 ± 12.94	83.52 ± 19.99	0.0647
**2 Days**	58.70 ± 7.94^{B}	71.70 ± 12.78^{B}	228.06 ± 49.68^{A}	0.0001
**3 Days**	58.55 ± 10.06^{B}	54.11 ± 11.16^{B}	262.10 ± 44.08^{A}	0.0001
**5 Days**	47.71 ± 6.64^{B}	59.95 ± 16.01^{B}	273.94 ± 48.00^{A}	0.0001
**7 Days**	70.33 ± 17.19^{B}	54.24 ± 12.08^{B}	264.15 ± 50.64^{A}	0.0001
**10 Days**	55.94 ± 11.63^{B}	59.64 ± 18.72^{B}	345.51 ± 41.54^{A}	0.0001
**15 Days**	75.89 ± 20.04^{B}	41.76 ± 8.03^{B}	369.29 ± 64.22^{A}	0.0001
**30 Days**	79.14 ± 20.42^{B}	123.29 ± 32.93^{B}	292.03 ± 33.13^{A}	0.0001

A, B - different letters on the line mean statistically significant difference compared with Tukey's test.

Table S8 – Mean values and standard error of the mean obtained for the Total Capacity to Bind Iron to Transferrin (TIBIC), expressed in µL/dL, of Nellore calves during the first month of life and grouped according to type of birth (elective cesarean section without induction of delivery, elective cesarean section with labor induction and spontaneous vaginal calving).

		**TIBIC µL/dL**		
**Time of life (hours or days)**	Elective cesarean section without inducing labor	Elective cesarean section with labor induction	Spontaneous vaginal calving	*P-Values*
**0 Hours**	307.96 ± 10.84^{B}	321.93 ± 22.86^{AB}	478.32 ± 32.01^{A}	0.0001
**3 Hours**	269.12 ± 12.84^{B}	284.72 ± 6.76^{AB}	466.46 ± 27.92^{A}	0.0001
**6 Hours**	240.80 ± 13.15^{B}	255.14 ± 11.38^{AB}	423.17 ± 22.78^{A}	0.0001
**12 Hours**	232.17 ± 14.17^{B}	206.50 ± 29.07^{B}	412.00 ± 21.80^{A}	0.0001
**1 Day**	223.14 ± 12.53^{B}	220.02 ± 28.17^{B}	399.42 ± 28.84^{A}	0.0001
**2 Days**	246.90 ± 12.94^{B}	235.12 ± 34.52^{B}	434.66 ± 24.96^{A}	0.0001
**3 Days**	278.35 ± 21.37^{B}	226.25 ± 42.01^{B}	470.10 ± 28.73^{A}	0.0001
**5 Days**	295.98 ± 16.97^{B}	187.20 ± 38.03^{B}	526.14 ± 23.76^{A}	0.0001
**7 Days**	329.33 ± 20.21^{B}	238.40 ± 41.16^{B}	578.25 ± 18.79^{A}	0.0001
**10 Days**	344.94 ± 23.27^{B}	271.10 ± 42.63^{B}	616.91 ± 25.38^{A}	0.0001
**15 Days**	353.92 ± 21.01^{B}	250.83 ± 49.87^{B}	612.59 ± 24.43^{A}	0.0001
**30 Days**	394.07 ± 28.71^{B}	378.47 ± 70.53^{B}	590.43 ± 36.53^{A}	0.0080

A, B - different letters on the line mean statistically significant difference compared with Tukey's test.

Table S9 – Mean values and standard error of the mean obtained for the Transferrin Saturation Index (TSI), expressed in %, of Nellore calves during the first month of life and grouped according to type of birth (elective cesarean section without labor induction, elective cesarean section with labor induction and spontaneous vaginal calving).

		**TSI %**		
**Time of life (hours or days)**	Elective cesarean section without inducing labor	Elective cesarean section with labor induction	Spontaneous vaginal calving	*P-Values*
**0 Hours**	51.64 ± 5.28^{B}	49.88 ± 2.15^{B}	14.24 ± 1.96^{A}	0.0001
**3 Hours**	48.17 ± 5.74^{B}	43.48 ± 4.20^{AB}	14.60 ± 1.51^{A}	0.0002
**6 Hours**	31.71 ± 4.55	32.71 ± 4.73	13.42 ± 1.60	0.0114
**12 Hours**	21.14 ± 2.55^{AB}	32.41 ± 3.72^{B}	10.24 ± 1.51^{A}	0.0008
**1 Day**	17.06 ± 2.82	35.86 ± 3.35	21.28 ± 5.64	0.0293
**2 Days**	24.22 ± 3.39^{B}	41.61 ± 3.39^{AB}	51.75 ± 9.83^{A}	0.0092
**3 Days**	21.56 ± 3.80^{B}	36.70 ± 5.79^{AB}	56.76 ± 9.65^{A}	0.0023
**5 Days**	16.99 ± 2.63^{B}	28.99 ± 5.19^{AB}	52.00 ± 9.33^{A}	0.0009
**7 Days**	21.86 ± 4.70	25.04 ± 3.72	44.26 ± 7.77	0.0291
**10 Days**	17.12 ± 3.64^{B}	24.09 ± 4.84^{AB}	56.19 ± 6.51^{A}	0.0001
**15 Days**	22.26 ± 5.15^{B}	28.46 ± 5.12^{AB}	59.76 ± 9.88^{A}	0.0028
**30 Days**	19.26 ± 4.36^{B}	57.97 ± 6.95^{A}	49.48 ± 4.94^{A}	0.0001

A, B - different letters on the line mean statistically significant difference compared with Tukey's test.
